# Plant-Based Diets and Disease Progression in Men With Prostate Cancer

**DOI:** 10.1001/jamanetworkopen.2024.9053

**Published:** 2024-05-01

**Authors:** Vivian N. Liu, Erin L. Van Blarigan, Li Zhang, Rebecca E. Graff, Stacy Loeb, Crystal S. Langlais, Janet E. Cowan, Peter R. Carroll, June M. Chan, Stacey A. Kenfield

**Affiliations:** 1 Department of Epidemiology and Biostatistics, University of California, San Francisco; 2 Menwell Limited, London, England, United Kingdom; 3 Department of Urology, University of California, San Francisco; 4 Department of Medicine, University of California, San Francisco; 5 Department of Urology and Population Health, New York University and Manhattan Veterans Affairs, New York; 6 Real World Solutions, IQVIA, Durham, North Carolina

## Abstract

**Importance:**

Plant-based diets are associated with many health and environmental benefits, including primary prevention of fatal prostate cancer, but less is known about postdiagnostic plant-based diet patterns in individuals with prostate cancer.

**Objective:**

To examine whether postdiagnostic plant-based dietary patterns are associated with risk of prostate cancer progression and prostate cancer–specific mortality.

**Design, Setting, and Participants:**

This longitudinal observational cohort study included men with biopsy-proven nonmetastatic prostate cancer (stage ≤T3a) from the diet and lifestyle substudy within the Cancer of the Prostate Strategic Urologic Research Endeavor (CaPSURE) enrolled at 43 urology practices across the US from 1999 to 2018. Participants completed a comprehensive diet and lifestyle questionnaire (including a validated food frequency questionnaire [FFQ]) between 2004 and 2016. Data were analyzed from August 2022 to April 2023.

**Exposures:**

Overall plant-based diet index (PDI) and healthful plant-based diet index (hPDI) scores were calculated from the FFQ.

**Main Outcomes and Measures:**

The primary outcome was prostate cancer progression (recurrence, secondary treatment, bone metastases, or prostate cancer–specific mortality). The secondary outcome was prostate cancer–specific mortality.

**Results:**

Among 2062 participants (median [IQR] age, 65.0 [59.0-70.0] years), 61 (3%) identified as African American, 3 (<1%) identified as American Indian or Alaska Native, 9 (<1%) identified as Asian or Pacific Islander, 15 (1%) identified as Latino, and 1959 (95%) identified as White. Median (IQR) time from prostate cancer diagnosis to FFQ was 31.3 (15.9-62.0) months after diagnosis. During a median (IQR) follow-up of 6.5 (1.3-12.8) years after the FFQ, 190 progression events and 61 prostate cancer–specific mortality events were observed. Men scoring in the highest vs lowest quintile of PDI had a 47% lower risk of progression (HR, 0.53; 95% CI, 0.37-0.74; *P* for trend = .003). The hPDI was not associated with risk of progression overall. However, among 680 individuals with Gleason grade 7 or higher at diagnosis, the highest hPDI quintile was associated with a 55% lower risk of progression compared with the lowest hPDI quintile (HR 0.45; 95% CI, 0.25-0.81; *P* for trend = .01); no association was observed in individuals with Gleason grade less than 7.

**Conclusions and Relevance:**

In this cohort study of 2062 men with prostate cancer, higher intake of plant foods after prostate cancer diagnosis was associated with lower risk of cancer progression. These findings suggest nutritional assessment and counseling may be recommended to patients with prostate cancer to help establish healthy dietary practices and support well-being and overall health.

## Key Points

**Question:**

What is the association between postdiagnostic plant-based dietary patterns and risk of prostate cancer progression?

**Findings:**

In a cohort study of 2062 men diagnosed with nonmetastatic prostate cancer, individuals with the highest intake of plant foods in the overall plant-based diet index had lower risk of prostate cancer progression compared with those with the lowest intake.

**Meaning:**

These findings suggest that consuming a primarily plant-based diet may be associated with better prostate cancer–specific health outcomes among men with prostate cancer.

## Teaser

This cohort study examines whether postdiagnostic plant-based dietary patterns are associated with risk of prostate cancer progression or prostate cancer–specific mortality in men with nonmetastatic prostate cancer.

## Introduction

Prostate cancer is the second most common cancer among men in the US. Plant-based diets (ie, diets incorporating a greater proportion of one’s daily caloric intake from plant sources) are increasingly popular^1^ and have nutritional benefits among people diagnosed with various chronic diseases, including prostate cancer.^2,3,4,5,6^ Current dietary recommendations for patients with cancer and the general population emphasize a plant-based diet high in fruits, vegetables, and whole grains.^7^

Yet, little is known about plant-based dietary patterns and prostate cancer–specific clinical outcomes after diagnosis. Many studies have reported that greater intake of individual plant-based foods (eg, cruciferous vegetables, cooked tomatoes, vegetable fats) is associated with lower risk of prostate cancer recurrence or mortality,^8,9,10,11,12,13,14^ but single dietary factors in isolation may not accurately capture the health effects of whole diets.^15^ Given the increasing interest in plant-based food at the population level, examining whether plant-based dietary patterns are associated with disease outcomes has important implications for public health.

Therefore, we evaluated postdiagnosis intake of plant-based foods in relation to clinical outcomes among patients with prostate cancer. We focused on 2 plant-based diet indices: the overall plant-based diet index (PDI) and healthful plant-based index (hPDI). These indices were developed in 2016 in 3 large cohort studies and have been associated with risk of diabetes, coronary heart disease, and total mortality.^2,3^ In addition, in the Health Professionals Follow-Up Study, they were associated with a lower risk of fatal prostate cancer and better scores for quality of life among men diagnosed with prostate cancer.^16,17^ We hypothesized that greater consumption of plant foods in both indices would be associated with lower risk of prostate cancer progression and prostate cancer–specific mortality.

## Methods

### Study Design

This cohort study was conducted in accordance with the Belmont Report and the US Common Rule under local institutional review board approval. All participants provided written informed consent. This study followed the Strengthening the Reporting of Observational Studies in Epidemiology (STROBE) reporting guideline for cohort studies. We used data from the Cancer of the Prostate Strategic Urologic Research Endeavor (CaPSURE), a longitudinal observational study of 15 310 men with biopsy-proven prostate cancer. Participants were enrolled concurrently from 43 urology practices across the US from 1999 to 2018.^18^ Participating urologists collected data on clinical and pathological factors, treatments, and recurrence.

### Study Population

A subset of individuals from the CaPSURE study were invited to participate in the CaPSURE Diet and Lifestyle substudy, consisting of a comprehensive diet and lifestyle questionnaire with a validated food frequency questionnaire (FFQ). Invitations to participate in the substudy were sent to all active participants at 3 time points between 2004 and 2016. If more than 1 survey was completed, we used the first completed FFQ to standardize exposure assessment.

Participants with a last clinical follow-up or documented progression (defined as recurrence, secondary treatment, bone metastases, or prostate cancer–specific mortality) prior to completion of a diet and lifestyle questionnaire were excluded. To reduce measurement error in usual diet,^19,20^ individuals with an extreme or unknown caloric intake (<800 kcal/d or >4200 kcal/d) or missing 70 or more FFQ items were excluded. Lastly, individuals with unknown clinical T-stage or T-stage T3a or higher were excluded. For the prostate cancer–specific mortality analyses (secondary outcome), we included individuals who had documented progression prior to completing the FFQ, since these people were still at risk for prostate cancer–specific mortality.

### Dietary Assessment

Dietary data were collected with a validated semiquantitative FFQ based on the one used to develop the diet indices.^21^ Participants were asked on average, how often (ranging from never or <1 serving/mo to ≥6 servings/d) they consumed a standard portion size of approximately 140 distinct foods and beverages in the past year.

To compute the plant-based diet indices, 18 food groups were created based on nutrients and culinary similarities, then classified into 3 larger categories of 7 healthful plant foods (whole grains, fruits, vegetables, nuts, legumes, vegetable oils, and tea and coffee), 5 unhealthful plant foods (fruit juices, sugar-sweetened beverages, refined grains, potatoes, and sweets or desserts), and 6 animal foods (animal fats, dairy, eggs, fish and seafood, meat, and miscellaneous animal-based foods).^2^ Intakes of the 18 food groups (servings per day) were ranked into quintiles (Qs). For PDI, greater amounts of both the healthful and unhealthful plant groups were given higher scores (ie, Q1 indicates a score 1; Q2, 2; Q3, 3; Q4, 4; Q5, 5), whereas animal food groups were given lower scores (ie, Q5 indicates a score of 1; Q4, 2; Q3, 3; Q2, 4; Q1, 5). For hPDI, the healthful plant food group was given increasing scores, while unhealthful plant food and animal food groups were given decreasing scores. Scores for the 18 groups were summed, ranging from 18 (lowest plant intake) to 90 (highest plant intake).

### Outcome Ascertainment

Our primary outcome was time to prostate cancer progression, a composite outcome comprised of biochemical recurrence, secondary treatment, bone metastases, or death attributed to prostate cancer. If participants had multiple progression events, the first reported date was used. Biochemical recurrence was defined as either 2 consecutive prostate-specific antigen (PSA) levels at least 0.2 ng/mL (to convert to micrograms per liter, multiply by 1) after radical prostatectomy or 2 consecutive PSA levels at least 2.0 ng/mL greater than the postradiation nadir.^22^ Date of recurrence was recorded as the date of the second elevated PSA. Secondary treatment was defined as any treatment that started at least 6 months after primary treatment completion. Bone metastases were attributed to prostate cancer if a urologist reported prostate cancer progression to bone or advancement to stage M1b, the patient had a positive bone scan, or the patient underwent radiation to treat bone metastases. Cause of death was determined by the registry data coordinating center and confirmed by state death certificate or the National Center for Health Statistics National Death Index. For analyses focused on clinical progression, participants were administratively censored at their last known clinical follow-up date up until January 31, 2019.

Prostate cancer–specific mortality was our secondary outcome, given the small number of prostate cancer–specific mortality events in this cohort. For these analyses, participants with a last known clinical follow-up date beyond December 30, 2020 (last National Death Index search), were administratively censored on December 30, 2020.

### Statistical Analysis

Medians and IQRs were calculated for continuous patient and clinical characteristics, and number and percentage was calculated for categorical characteristics, overall and by quintile, of the index scores. Median and IQR consumption of the 18 individual food groups were also computed in servings per day.

Pearson correlation coefficient was used to describe the correlation between the PDI and hPDI. We used Cox proportional hazards models and cause-specific models to evaluate the associations between the PDIs and the risk of prostate cancer progression and prostate cancer–specific mortality, respectively. All models were clustered by CaPSURE clinical site, with robust standard errors used to calculate 95% CIs. Simple models were adjusted for days from diagnosis to FFQ, age at diagnosis (years), year of diagnosis, and total energy intake (kcal). In the full multivariable models, we additionally adjusted for clinical T-stage (T1, T2, T3a), Gleason score (<7, 7, >7), and PSA (≤6, >6 to 10, >10 ng/mL) at diagnosis, primary treatment (radical prostatectomy, radiation, hormonal therapy, watchful waiting or active surveillance, other); self-reported race and ethnicity; smoking status (current, former, never); walking pace (<2, 2 to <3, 3 to <4, >4 mph, unable), and body mass index. Race and ethnicity were self reported and categorized as African American, Asian or Pacific Islander, Latino, multiple, Native American, White, and unknown; race and ethnicity were used in adjustment as White or other. Race and ethnicity were included in analysis because they are strongly associated with prostate cancer outcomes. Additional covariates, including diabetes, family history of prostate cancer, household income, education level, height, alcohol use, multivitamin use, calcium supplement use, and selenium supplement use, were considered but did not meaningfully change results, so they were not included in final models. Log-minus-log plots and Schoenfeld tests were used to test the proportional hazards assumption, and Martingale residuals and smoothing were used to assess the linearity of predictors assumption. Contrast analyses were used to assess for linear trends.

In secondary analyses, we examined each of the 3 food groups comprising the indices (healthful, unhealthful, animal) in association with prostate cancer progression. We also explored potential modification by walking pace (<3 vs ≥3 mph), age (<65 years vs ≥65 years), stage (T1, T2, or T3a), PSA (<6, 6-10, or >10 ng/mL), and Gleason grade at diagnosis (<7, ≥7). To evaluate the significance of interactions between the PDI or hPDI and these variables, we used separate multivariable models including cross product terms between the index and effect modifier of interest. We then used likelihood ratio tests to compare models with and without these interaction terms. For the covariates that demonstrated statistically significant interactions, stratified subgroup analyses were performed.

All analyses were performed in Stata software version 17 (StataCorp) using a 2-sided α = .05 to assess statistical significance. Data were analyzed from August 2022 to April 2023.

## Results

A total of 2891 participants completed at least 1 survey, and a total of 2062 participants (median [IQR] age at diagnosis, 65.0 [59.0-70.0] years) met the inclusion criteria; 61 (3%) identified as African American, 3 (<1%) identified as American Indian or Alaska Native, 9 (<1%) identified as Asian or Pacific Islander, 15 (1%) identified as Latino, and 1959 (95%) identified as White. Participant characteristics, overall and by quintile of the PDI and hPDI at baseline, are displayed in Table 1. Compared with participants in the lowest PDI and hPDI quintile, participants in the highest quintile of PDI and hPDI had a faster walking pace, lower body mass index, and lower diagnostic PSA and were less likely to smoke (Table 1). Participants in the highest PDI quintile consumed more calories than those in the lowest PDI quintile, whereas individuals in hPDI Q5 consumed fewer calories and were younger than those in Q1. Characteristics for participants in the prostate cancer–specific mortality analyses were nearly identical (eTable 1 in Supplement 1). PDI and hPDI scores were moderately positively correlated (*r* = 0.34; *P* < .001). PDI scores ranged from 27 to 76, and hPDI scores ranged from 29 to 84.

**Table 1.** Patient and Clinical Characteristics of Men With Localized Prostate Cancer, Overall and Stratified by Quintiles of PDI and hPDI Scores

Characteristic	Patients, No. (%)											
	Total	PDI					hPDI					
		Q1 (lowest)	Q2	Q3	Q4	Q5 (highest)	Q1 (lowest)	Q2	Q3	Q4	Q5 (highest)
Participants, No.	2062	462	412	365	480	343	413	421	496	332	400
Age, median (IQR), y	65.0 (59.0-70.0)	64.0 (58.0-69.0)	65.0 (59.0-70.0)	65.0 (60.0-70.0)	65.0 (60.0-70.0)	64.0 (58.0-70.0)	66.0 (59.0-71.0)	65.0 (59.0-71.0)	65.0 (59.0-70.0)	64.0 (60.0-69.0)	63.0 (58.0-68.0)
BMI, median (IQR)	26.9 (24.7-29.6)	27.9 (25.8-30.6)	27.1 (24.9-29.6)	27.1 (25.0-29.8)	26.5 (24.4-29.3)	25.8 (23.8-28.2)	27.3 (25.1-30.0)	27.3 (25.1-29.8)	26.9 (24.5-29.4)	26.6 (24.7-29.6)	26.2 (24.4-28.9)
PSA, median (IQR), ng/mL	5.6 (4.4-7.9)	5.8 (4.4-8.1)	5.8 (4.5-8.5)	5.5 (4.3-7.7)	5.5 (4.3-7.3)	5.4 (4.4-7.4)	5.8 (4.4-8.2)	5.7 (4.5-7.9)	5.7 (4.3-8.3)	5.3 (4.3-7.4)	5.3 (4.3-7.6)
Calorie intake, median (IQR), kcal	1922 (1526-2340)	1640 (1298-2016)	1856 (1468-2269)	1840 (1506-2261)	2020 (1656-2490)	2278 (1899-2738)	2296 (1967-2679)	1950 (1575-2342)	1834 (1459-2287)	1725 (1425-2166)	1725 (1392-2119)
Alcohol intake, median (IQR), servings/d	0.4 (0.0-1.2)	0.3 (0.0-1.2)	0.4 (0.0-1.2)	0.4 (0.1-1.2)	0.4 (0.0-1.1)	0.5 (0.1-1.2)	0.2 (0.0-1.0)	0.2 (0.0-1.1)	0.4 (0.0-1.3)	0.4 (0.0-1.2)	0.6 (0.1-1.3)
Wine intake, median (IQR), servings/d	0.1 (0.0-0.4)	0.0 (0.0-0.1)	0.1 (0.0-0.3)	0.1 (0.0-0.3)	0.1 (0.0-0.5)	0.1 (0.0-0.8)	0.0 (0.0-0.1)	0.0 (0.0-0.1)	0.1 (0.0-0.4)	0.1 (0.0-0.4)	0.1 (0.0-0.8)
Race and ethnicity^a^											
African American	61 (3)	10 (2)	13 (3)	9 (2)	21 (4)	8 (2)	8 (2)	11 (3)	11 (2)	17 (5)	14 (4)
Asian or Pacific Islander	9 (<1)	1 (<1)	2 (1)	2 (1)	2 (<1)	2 (1)	0	4 (1)	1 (<1)	1 (<1)	3 (1)
Latino	15 (1)	3 (1)	3 (1)	1 (0.3)	3 (1)	5 (1)	1 (0.2)	0	4 (1)	3 (1)	7 (2)
Multiple	10 (1)	1 (<1)	7 (2)	0	2 (<1)	0	1 (<1)	0	3 (1)	4 (1)	2 (1)
Native American	3 (<1)	0	1 (<1)	1 (<1)	1 (<1)	0	0	2 (1)	0	0	1 (<1)
White	1959 (95)	446 (97)	384 (93)	351 (96)	450 (94)	328 (96)	401 (97)	404 (96)	475 (96)	307 (92)	372 (93)
Unknown	5 (<1)	1 (<1)	2 (<1)	1 (<1)	1 (<1)	0	2 (<1)	0	2 (<1)	0	1 (<1)
Smoking Status											
Never	918 (45)	179 (39)	176 (44)	162 (45)	225 (47)	176 (52)	174 (43)	195 (47)	220 (45)	127 (39)	202 (51)
Former	1011 (50)	242 (53)	208 (51)	177 (49)	235 (49)	149 (44)	205 (50)	191 (46)	242 (50)	188 (57)	185 (46)
Current	106 (5)	34 (7)	20 (5)	21 (6)	17 (4)	14 (4)	29 (7)	30 (7)	24 (5)	12 (4)	11 (3)
Walking pace, mph											
Unable	19 (1)	8 (2)	2 (1)	5 (1)	2 (<1)	2 (1)	2 (<1)	3 (1)	5 (1)	6 (2)	3 (1)
<2 (easy)	307 (15)	67 (15)	74 (18)	49 (14)	73 (15)	44 (13)	78 (19)	67 (16)	80 (17)	42 (13)	40 (10)
2 to <3 (reference range)	1017 (50)	244 (55)	206 (51)	189 (53)	219 (46)	159 (47)	217 (54)	232 (57)	232 (48)	173 (53)	163 (41)
3 to <4 (brisk)	595 (29)	116 (26)	109 (27)	99 (28)	163 (34)	108 (32)	91 (23)	99 (24)	147 (30)	97 (30)	161 (40)
≥4 (fast)	84 (4)	12 (3)	13 (3)	16 (4)	17 (4)	26 (8)	14 (3)	9 (2)	20 (4)	9 (3)	32 (8)
Family History of prostate cancer											
No	1650 (80)	377 (82)	337 (82)	291 (80)	380 (79)	265 (77)	340 (82)	339 (81)	380 (77)	276 (83)	315 (79)
Yes	412 (20)	85 (18)	75 (18)	74 (20)	100 (21)	78 (23)	73 (18)	82 (19)	116 (23)	56 (17)	85 (21)
Diabetes status											
No	1908 (93)	399 (86)	376 (91)	347 (95)	450 (94)	336 (98)	384 (93)	397 (94)	453 (91)	304 (92)	370 (92)
Yes	154 (7)	63 (14)	36 (9)	18 (5)	30 (6)	7 (2)	29 (7)	24 (6)	43 (9)	28 (8)	30 (8)
Gleason at diagnosis											
<7	1375 (67)	315 (69)	269 (66)	258 (71)	310 (65)	223 (65)	279 (69)	285 (68)	327 (67)	208 (63)	276 (69)
7	539 (26)	120 (26)	105 (26)	84 (23)	135 (28)	95 (28)	102 (25)	99 (24)	137 (28)	102 (31)	99 (25)
>7	131 (6)	22 (5)	33 (8)	21 (6)	32 (7)	23 (7)	26 (6)	34 (8)	27 (5)	21 (6)	23 (6)
T-Stage at diagnosis											
≤T1	1185 (57)	270 (58)	225 (55)	201 (55)	283 (59)	206 (60)	227 (55)	246 (58)	287 (58)	185 (56)	240 (60)
T2	858 (42)	188 (41)	183 (44)	161 (44)	193 (40)	133 (39)	180 (44)	170 (40)	206 (42)	146 (44)	156 (39)
T3a	19 (1)	4 (1)	4 (1)	3 (1)	4 (1)	4 (1)	6 (1)	5 (1)	3 (1)	1 (<1)	4 (1)
Primary treatment											
Radical prostatectomy	1249 (62)	277 (61)	249 (62)	221 (62)	279 (60)	223 (67)	232 (57)	262 (64)	303 (62)	202 (62)	250 (66)
Active surveillance or watchful waiting	124 (6)	14 (3)	22 (5)	29 (8)	36 (8)	23 (7)	17 (4)	11 (3)	33 (7)	22 (7)	41 (11)
RT or brachytherapy	448 (22)	115 (25)	94 (23)	79 (22)	98 (21)	62 (19)	106 (26)	100 (24)	100 (21)	76 (23)	66 (17)
Hormone therapy	105 (5)	28 (6)	20 (5)	12 (3)	29 (6)	16 (5)	26 (6)	27 (7)	26 (5)	14 (4)	12 (3)
Other	82 (4)	21 (5)	19 (5)	14 (4)	21 (5)	7 (2)	25 (6)	12 (3)	23 (5)	10 (3)	12 (3)
Multivitamin use											
Never	452 (22)	100 (22)	101 (25)	79 (22)	97 (20)	75 (22)	102 (25)	101 (24)	116 (24)	74 (22)	59 (15)
Former	396 (20)	97 (21)	72 (18)	66 (18)	94 (20)	67 (20)	66 (16)	84 (20)	108 (22)	66 (20)	72 (18)
Current	1181 (58)	256 (57)	229 (57)	214 (60)	283 (60)	199 (58)	233 (58)	229 (55)	268 (54)	189 (57)	262 (67)

Abbreviations: BMI, body mass index (calculated as weight in kilograms divided by height in meters squared); hPDI, healthful plant-based diet index; PDI, overall plant-based diet index; PSA, prostate specific antigen; Q, quintile.

SI conversion factor: To convert PSA to micrograms per liter, multiply by 1.

^a^ These are category labels participants were presented with on the survey (survey year: 2004).

Servings per day of individual dietary score components by lowest and highest quintile of PDI and hPDI are shown in Table 2. Participants in the highest vs lowest quintile, consumed a mean of approximately 1.9 additional servings of vegetables, 1.6 additional servings of fruit, 0.9 more servings of whole grains, 1.0 less serving of dairy, 0.4 less servings of animal fat, slightly less egg, and marginally less meat (Table 2).

**Table 2.** Median Servings per Day of Individual Food Components by Quintile of Each Index

Component	PDI						hPDI					
	Score^a^	Servings/d, median (IQR)					Score^a^	Servings/d, median (IQR)				
		Q1 (lowest)	Q2	Q3	Q4	Q5 (highest)		Q1 (lowest)	Q2	Q3	Q4	Q5 (highest)
Participants, No.	NA	462	412	365	480	343	NA	413	421	496	332	400
Whole grains	+	0.4 (0.1-0.9)	0.6 (0.3-1.0)	0.9 (0.4-1.3)	0.9 (0.6-1.5)	1.3 (0.9-2.4)	+	0.6 (0.2-1.0)	0.7 (0.3-1.3)	0.7 (0.3-1.2)	0.8 (0.5-1.4)	1.1 (0.6-1.9)
Fruits	+	0.8 (0.4-1.6)	1.3 (0.6-1.9)	1.4 (0.9-2.3)	1.9 (1.2-2.7)	2.4 (1.6-3.3)	+	1.2 (0.6-1.9)	1.4 (0.7-2.2)	1.4 (0.8-2.3)	1.7 (1.0-2.6)	2.1 (1.2-3.1)
Vegetables	+	2.2 (1.3-3.2)	2.5 (1.7-3.6)	2.8 (2.0-4.1)	3.3 (2.3-4.8)	4.1 (2.8-5.8)	+	2.4 (1.6-3.7)	2.5 (1.7-3.7)	2.7 (1.7-4.1)	3.2 (2.1-4.6)	3.8 (2.6-5.3)
Nuts	+	0.1 (0.0-0.1)	0.1 (0.1-0.4)	0.1 (0.1-0.4)	0.4 (0.1-0.7)	0.4 (0.1-1.0)	+	0.1 (0.0-0.1)	0.1 (0.0-0.4)	0.1 (0.1-0.4)	0.1 (0.1-0.8)	0.4 (0.1-1.0)
Legumes	+	0.1 (0.0-0.1)	0.1 (0.0-0.1)	0.1 (0.1-0.1)	0.1 (0.1-0.2)	0.1 (0.1-0.4)	+	0.1 (0.0-0.1)	0.1 (0.1-0.1)	0.1 (0.1-0.1)	0.1 (0.1-0.2)	0.1 (0.1-0.4)
Vegetable oils	+	0.1 (0.0-0.1)	0.1 (0.0-0.4)	0.1 (0.1-0.4)	0.1 (0.1-0.4)	0.4 (0.1-0.8)	+	0.1 (0.0-0.1)	0.1 (0.0-0.4)	0.1 (0.1-0.4)	0.1 (0.1-0.4)	0.4 (0.1-0.8)
Tea/coffee	+	1.1 (0.5-2.5)	1.6 (0.9-2.6)	2.2 (1.0-2.8)	2.4 (1.0-2.9)	2.5 (1.1-3.2)	+	1.4 (0.8-2.6)	1.6 (0.8-2.6)	1.8 (0.9-2.6)	2.1 (1.0-2.9)	2.5 (1.0-3.3)
Fruit juice	+	0.1 (0.0-0.8)	0.4 (0.1-1.0)	0.4 (0.1-1.0)	0.8 (0.2-1.1)	1.0 (0.4-1.2)	−	0.9 (0.2-1.1)	0.6 (0.1-1.1)	0.4 (0.1-1.0)	0.4 (0.1-1.0)	0.1 (0.0-0.8)
Refined grains	+	1.0 (0.6-1.6)	1.3 (0.8-1.9)	1.3 (0.9-1.9)	1.5 (0.9-2.1)	1.8 (1.3-2.5)	−	2.1 (1.5-2.8)	1.5 (1.0-2.1)	1.3 (0.8-1.8)	1.1 (0.6-1.6)	1.0 (0.6-1.4)
Total potatoes	+	0.3 (0.1-0.6)	0.4 (0.2-0.6)	0.4 (0.2-0.6)	0.5 (0.2-0.7)	0.6 (0.3-0.8)	−	0.6 (0.5-1.0)	0.5 (0.3-0.7)	0.4 (0.2-0.6)	0.3 (0.1-0.5)	0.2 (0.1-0.4)
Sugar-sweetened beverages	+	0.0 (0.0-0.1)	0.1 (0.0-0.4)	0.1 (0.0-0.3)	0.1 (0.0-0.4)	0.1 (0.0-0.4)	−	0.4 (0.1-0.9)	0.1 (0.0-0.4)	0.1 (0.0-0.3)	0.0 (0.0-0.1)	0.0 (0.0-0.1)
Sweets/desserts	+	0.6 (0.3-1.1)	0.9 (0.5-1.7)	1.0 (0.5-1.6)	1.2 (0.6-2.1)	1.6 (0.9-2.6)	−	1.7 (1.0-2.8)	1.2 (0.7-2.0)	1.0 (0.5-1.7)	0.7 (0.4-1.3)	0.6 (0.3-1.1)
Animal fat	−	0.8 (0.4-1.0)	0.6 (0.2-1.0)	0.5 (0.1-1.0)	0.4 (0.1-0.9)	0.4 (0.1-0.8)	−	1.0 (0.4-2.0)	0.8 (0.3-1.0)	0.4 (0.1-0.9)	0.4 (0.1-0.8)	0.1 (0.0-0.4)
Dairy	−	3.2 (1.7-5.4)	3.1 (1.7-5.6)	2.5 (1.5-4.4)	2.6 (1.5-4.8)	2.2 (1.4-3.4)	−	4.0 (2.3-6.4)	3.1 (1.8-5.4)	2.6 (1.6-4.4)	2.0 (1.3-3.3)	1.8 (1.1-3.4)
Egg	−	0.4 (0.1-0.6)	0.4 (0.1-0.4)	0.4 (0.1-0.4)	0.3 (0.1-0.4)	0.1 (0.1-0.4)	−	0.4 (0.1-0.6)	0.4 (0.1-0.4)	0.4 (0.1-0.4)	0.1 (0.1-0.4)	0.1 (0.1-0.4)
Fish/seafood	−	0.3 (0.1-0.4)	0.3 (0.1-0.4)	0.2 (0.1-0.4)	0.2 (0.1-0.4)	0.3 (0.1-0.4)	−	0.3 (0.1-0.4)	0.2 (0.1-0.4)	0.2 (0.1-0.4)	0.3 (0.1-0.4)	0.3 (0.1-0.4)
Meat	−	1.3 (0.8-1.9)	1.2 (0.8-1.8)	1.1 (0.8-1.6)	1.1 (0.7-1.6)	1.1 (0.7-1.6)	−	1.6 (1.1-2.1)	1.4 (0.9-1.8)	1.1 (0.8-1.6)	1.0 (0.6-1.5)	0.8 (0.6-1.2)
Miscellaneous animal	−	0.2 (0.1-0.5)	0.2 (0.1-0.5)	0.2 (0.1-0.4)	0.2 (0.1-0.4)	0.1 (0.1-0.3)	−	0.4 (0.2-0.6)	0.2 (0.1-0.5)	0.2 (0.1-0.4)	0.1 (0.1-0.3)	0.1 (0.1-0.1)

Abbreviations: +, positive; −, negative; hPDI, healthful plant-based diet index; NA, not applicable; PDI, overall plant-based diet index; Q, quintile.

^a^ Intakes of the 18 food groups (servings per day) were categorized into quintiles, and each quintile was assigned a score between 1 and 5. Depending on the index, the food groups were given positive (Q1 indicates a score of 1; Q2, 2; Q3, 3; Q4, 4; Q5, 5) or negative scores (Q5 indicates a score of 1; Q4, 2; Q3, 3; Q2, 4; Q1, 5). Scores for the 18 groups were summed, such that they ranged from 18 (lowest plant intake) to 90 (highest plant intake). Higher scores for both indices reflected higher plant intake.

Table 3 shows hazard ratios (HRs) and 95% CIs for associations between the PDIs and prostate cancer progression. Of 2062 participants who met the inclusion criteria for the primary end point analyses, we observed 190 progression events (170 participants with biochemical recurrence, 7 participants with bone metastases, and 13 deaths related to prostate cancer; there were no secondary treatment events that were not preceded by 1 of the other outcomes) as the first recorded event over a median (IQR) follow-up of 6.5 (1.3-12.8) years after FFQ completion. In the fully adjusted models, participants in the highest quintile of PDI had a 47% lower risk of progression compared with individuals in the lowest quintile (HR, 0.53; 95% CI, 0.37-0.74; *P* for trend = .003). In contrast, there was no evidence of an association with the hPDI (Q5 vs Q1: HR, 0.81; 95% CI, 0.54-1.20; *P* for trend = .34). There was no statistically significant difference in analysis in the healthful plant food group (Q5 vs Q1: HR_,_ 0.58; 95% CI, 0.34-1.00; *P* for trend = .08) (eTable 2 in Supplement 1). No associations were seen between the unhealthful plant nor animal food groups with risk of prostate cancer progression.

**Table 3.** Associations of PDI and hPDI With Risk of Prostate Cancer Progression or Prostate Cancer–Specific Mortality Among Men Initially Diagnosed With Nonmetastatic Prostate Cancer

Measure	Quintile					*P* value for trend
	1 (Lowest)	2	3	4	5 (Highest)	
**Progression (n = 2062)**						
PDI						
Events, No.	52	46	27	47	18	NA
Index Score, mean (SD)	45.17 (3.27)	50.59 (1.07)	54.33 (0.73)	57.83 (1.24)	65.00 (3.09)	NA
Model 1, HR (95% CI)^a^	1 [Reference]	1.03 (0.74-1.43)	0.61 (0.35-1.05)	0.85 (0.50-1.42)	0.48 (0.33-0.69)	<.001
Model 2, HR (95% CI)^b^	1 [Reference]	1.02 (0.69-1.52)	0.74 (0.44-1.26)	0.98 (0.60-1.60)	0.53 (0.37-0.74)	.003
hPDI						
Events, No.	51	43	46	20	30	NA
Index Score, mean (SD)	42.18 (3.77)	49.74 (1.31)	54.89 (1.45)	58.75 (0.91)	66.90 (4.29)	NA
Model 1, HR (95% CI)^a^	1 [Reference]	0.86 (0.67-1.12)	0.90 (0.63-1.27)	0.64 (0.40-1.02)	0.77 (0.54-1.10)	.11
Model 2, HR (95% CI)^b^	1 [Reference]	0.78 (0.60-1.01)	0.94 (0.63-1.39)	0.68 (0.39-1.18)	0.81 (0.54-1.20)	.34
**Prostate cancer–specific mortality (n = 2274)**						
PDI						
Events, No.	22	12	6	12	9	NA
Index Score, mean (SD)	44.64 (3.55)	50.67 (1.15)	53.83 (0.75)	57.92 (1.16)	64.00 (2.50)	NA
Model 1, HR (95% CI)^a^	1 [Reference]	0.55 (0.34-0.87)	0.28 (0.12-0.68)	0.41 (0.23-0.75)	0.50 (0.21-1.16)	.09
Model 2, HR (95% CI)^b^	1 [Reference]	0.49 (0.26-0.95)	0.44 (0.18-1.13)	0.33 (0.14-0.78)	0.53 (0.17-1.66)	.16
hPDI						
Events, No.	15	18	14	5	9	NA
Index Score, mean (SD)	41.93 (4.01)	49.61 (1.33)	54.29 (1.49)	59.80 (1.10)	67.67 (3.46)	NA
Model 1, HR (95% CI)^a^	1 [Reference]	1.24 (0.46-3.31)	0.93 (0.43-2.01)	0.52 (0.18-1.49)	0.89 (0.39-2.06)	.23
Model 2, HR (95% CI)^b^	1 [Reference]	1.47 (0.52-4.17)	0.83 (0.34-2.05)	0.94 (0.31-2.88)	0.84 (0.28-2.56)	.52

Abbreviations: hPDI, healthful plant-based diet index; HR, hazard ratio; NA, not applicable; PDI, overall plant-based diet index.

^a^ Adjusted for days diagnosed to first questionnaire (continuous), age diagnosed (continuous), year diagnosed (continuous), total energy intake (continuous, kilocalories per day), and CaPSURE clinical site.

^b^ Additionally adjusted for T-stage at diagnosis (T1, T2, T3a); Gleason score at diagnosis (<7, 7, >7); PSA at diagnosis (≤6 ng/mL, >6 to 10 ng/mL, >10 ng/mL [to convert to micrograms per liter, multiply by 1]); primary treatment (radical prostatectomy, radiation, hormonal therapy, watchful waiting or active surveillance, other); self-reported race and ethnicity (White, other [African American, Asian or Pacific Islander, Latino, multiple, Native American, or unknown]); smoking status (current, former, never); walking pace (<2 mph, 2 to <3 mph, 3 to <4 mph, >4 mph, unable); and body mass index (continuous).

The sample for our secondary analysis of prostate cancer–specific mortality included 2274 participants, with 61 prostate cancer–specific deaths and 302 other deaths. While there were no statistically significant associations between either dietary index and risk of prostate cancer–specific mortality (Table 3), CIs were too wide to draw meaningful conclusions from point estimates (Q4 vs Q1: HR, 0.33; 95% CI, 0.14-0.78; Q5 vs Q1: HR, 0.53; 95% CI, 0.17-1.66; *P* for trend = .16).

For both indices, we found no evidence of interactions between age, PSA, stage at diagnosis, or walking pace. For hPDI, Gleason grade at diagnosis was associated with modifying the association of hPDI with prostate cancer progression (*P* for interaction = .03). Among participants with Gleason grade 7 or higher, participants in the highest quintile had a 55% lower risk of progression compared with the lowest quintile (HR, 0.45; 95% CI, 0.25-0.81; *P* for trend = .01) (eTable 3 in Supplement 1). There was no statistically significant association in individuals with Gleason grade less than 7. We did not detect association modification by any factors for the associations of the indices with prostate cancer–specific mortality.

## Discussion

This longitudinal cohort study investigated associations of plant-based dietary patterns after a diagnosis of localized prostate cancer with risk of prostate cancer progression. We did not evaluate the unhealthful plant-based diet index, as it would not be recommended for improving health outcomes. We observed an association whereby individuals who scored the highest on the overall PDI had lower risk of prostate cancer progression compared with those who scored the lowest.

Our findings align with previous reports that plant-based diets may improve prostate cancer outcomes. For example, in a study that evaluated PDI in association with risk of incident prostate cancer (47 243 men followed up for 28 years), Loeb et al^16^ reported that a higher PDI score was associated with 19% lower risk of incident prostate cancer that went on to be fatal (HR, 0.81; 95% CI, 0.64-1.01; *P* for trend = .04).

Our results contribute to the evolving body of research indicating the positive associations of plant-based diets with health outcomes. The PDI and its subindices were originally developed by Satija et al^2,3^ to evaluate the associations of PDI with type 2 diabetes and coronary heart disease. Satija et al^2,3^ found inverse associations between the overall PDI and hPDI for both outcomes. For PDI, other studies have observed a lower risk for diabetes,^5^ cardiovascular disease risk,^23^ cardiovascular mortality,^6,23^ and total mortality.^6,23^ For hPDI, studies have reported lower risk for diabetes,^5^ cardiovascular disease risk,^4^ cardiovascular mortality,^6^ and total mortality.^4,6^ These results are important in the context of localized prostate cancer, where men are more likely to die from these chronic diseases than their cancer.

We did not observe statistically significant associations for hPDI. Inconsistencies between the hPDI and overall PDI have been noted by others as well. In a 2022 study, Loeb et al^16^ observed associations between PDI and risk of developing fatal prostate cancer, whereas associations for hPDI were only seen for risk of developing localized prostate cancer. A study by Kim et al^23^ also reported statistically significant associations of PDI, but not hPDI, with risk of CVD.^23^ It may be that because the distribution of hPDI was relatively compressed compared with the distribution of PDI, the variance of the estimator increased corresponding with the association of hPDI. There were modest differences in servings per day between highest and lowest categories of fruit juice, refined grains, and sweets and desserts—all of which are categorized with equal weighting into the unhealthful food group and run in opposite directions for the PDI vs hPDI. Moreover, when looking at the healthful, unhealthful, and animal components of the subindices separately, there was no association with the unhealthful component with prostate cancer progression. Perhaps classifying some of these unhealthful plant foods as “bad actors” in the absence of an established detrimental association specifically with prostate cancer outcomes have attenuated the findings for hPDI. For PDI, we observed that people in Q5 (compared with Q1) consumed a mean of an additional 0.9 to 1.9 servings per day of healthful plant foods (particularly vegetables, fruits, and whole grains), while they consumed 0.3 to 1.0 fewer servings per day of animal products (particularly dairy, animal fat, and egg). These particular healthful foods have been associated with reduced risk in prostate cancer outcomes.^24,25,26^ While the similar consumption of fish and seafood, meat, and miscellaneous animal products among the extreme quintiles were unexpected, these results suggest that slightly reducing intake of animal products and placing more emphasis on more nutrient-dense plant-based foods may be advantageous.

Previous studies suggest several mechanisms through which plant-based diets may improve prostate cancer outcomes. Fruits and vegetables contain a variety of phytochemicals, including antioxidants and anti-inflammatory compounds, that have been shown to protect against prostate cancer.^24,26,27^ Plant foods are also a source of dietary fiber, which may promote satiety and regulate blood glucose levels.^28^ In addition, animal-based foods (including meat and dairy) have been associated with increased exposure to potentially harmful substances, such as hormones and heterocyclic amines.^29,30,31^ High intake of red and processed meats has been associated with increased insulin resistance and insulin-like growth factor-1, which have been linked to increased prostate cancer risk and potentially mortality.^32,33,34,35,36,37^ Furthermore, milk and dairy (a primary source of insulin-like growth factor-1), have been associated with increased risk of prostate cancer^37,38,39,40,41^; whole milk, in particular, has been associated with increased risk of prostate cancer recurrence.^38^

### Limitations

There are several limitations to the study. First, measurement error is a known limitation of self-reported data, including nutritional information. However, the FFQ used in this cohort has been validated, and the dietary data were collected prior to events of progression. Therefore, we expect measurement error in dietary intakes to be comparable in participants who experienced an event and those who did not. Second, even participants in the highest quintile of PDI consumed meat and dairy products; therefore we are unable to assess the associations of fully plant-based diets (eg, vegan, vegetarian). Third, the CaPSURE registry also is comprised of primarily of college-educated White men, which limits generalizability. Fourth, given that this was an observational study, we could not control for any unknown or unmeasured confounders. Other healthy behaviors and social determinants of health may be common causes of consuming more plant-based food and risk of prostate cancer progression. However, adjustment for income and education did not alter associations. Additionally, we were unable to adjust for prediagnostic diet in this cohort, so cannot conclude that the results are independent of prediagnostic exposure. Conversely, this study has several notable strengths, including a well-characterized cohort with extensive clinical follow-up and detailed diet data, as well as being the first to examine PDI and oncologic outcomes after prostate cancer diagnosis, to our knowledge.

## Conclusions

The findings of this cohort study suggest that plant-based dietary patterns may be inversely associated with risk of prostate cancer progression, although future research and replication of our findings is needed. These data are consistent with prior research demonstrating the importance of dietary factors in overall health and well-being.

## Notes

Supplement 1. **eTable 1.** Patient and Clinical Characteristics of Men With Localized Prostate Cancer, Overall and Stratified by Quintiles of PDI and hPDI Scores **eTable 2.** Multivariable Associations Between Each of the Three Food Groups and Risk of Prostate Cancer Progression **eTable 3.** Multivariable Associations Between the hPDI and Risk of Prostate Cancer Progression Among Men Initially Diagnosed With Nonmetastatic Prostate Cancer, Stratified by Gleason Grade

Supplement 2.
Data Sharing Statement
