# Supplementary material for: Plant-Based Diets and Disease Progression in Men With Prostate Cancer
Source: JAMA Netw Open. 2024 May 1;7(5):e249053. doi: 10.1001/jamanetworkopen.2024.9053 (PMC11063803; doi:10.1001/jamanetworkopen.2024.9053)
Supplement: Supplement 1. — eTable 1. Patient and Clinical Characteristics of Men With Localized Prostate Cancer, Overall and Stratified by Quintiles of PDI and hPDI Scores eTable 2. Multivariable Associations Between Each of the Three Food Groups and Risk of Prostate Cancer Progression eTable 3. Multivariable Associations Between the hPDI and Risk of Prostate Cancer Progression Among Men Initially Diagnosed With Nonmetastatic Prostate Cancer, Stratified by Gleason Grade [file jamanetwopen-e249053-s001.pdf]

Supplemental Online Content

Liu VN, Van Blarigan EL, Zhang L, et al. Plant-based diets and disease progression in men with prostate cancer. *JAMA Netw Open*. 2024;7(5):e249053. doi:10.1001/jamanetworkopen.2024.9053

eTable 1. Patient and Clinical Characteristics of Men With Localized Prostate Cancer, Overall and Stratified by Quintiles of PDI and hPDI Scores
eTable 2. Multivariable Associations Between Each of the Three Food Groups and Risk of Prostate Cancer Progression
eTable 3. Multivariable Associations Between the hPDI and Risk of Prostate Cancer Progression Among Men Initially Diagnosed With Nonmetastatic Prostate Cancer, Stratified by Gleason Grade

This supplemental material has been provided by the authors to give readers additional information about their work.

© 2024 Liu VN et al. *JAMA Network Open.*

eTable 1: Patient and clinical characteristics of 2274 men with localized prostate cancer, overall and stratified by quintiles of overall plant-based diet index (PDI) and healthful plant-based diet index (hPDI) scores (1=lowest score, 5=highest score).

	**Total**	**Overall Plant-based Diet Index (PDI)**					**Healthful Plant-based Diet Index (hPDI)**				
		Quintile 1 (lowest)	Quintile 2	Quintile 3	Quintile 4	Quintile 5 (highest)	Quintile 1 (lowest)	Quintile 2	Quintile 3	Quintile 4	Quintile 5 (highest)
**Number of participants, N**	2274	498	457	404	543	372	464	460	543	359	448
**Age (years), median (IQR)**	64.0 (59.0, 69.0)	63.5 (58.0, 69.0)	64.0 (59.0, 69.0)	65.0 (59.0, 70.0)	64.0 (60.0, 69.0)	64.0 (59.0, 70.0)	65.0 (59.0, 70.0)	65.0 (59.0, 70.0)	64.0 (59.0, 69.0)	65.0 (60.0, 69.0)	63.0 (58.0, 68.0)
**BMI, median (IQR)**	26.9 (24.7, 29.8)	28.1 (25.6, 30.7)	27.1 (25.1, 29.8)	27.1 (25.0, 29.8)	26.6 (24.4, 29.3)	25.7 (23.7, 28.0)	27.3 (25.1, 30.0)	27.3 (25.1, 30.0)	27.0 (24.7, 29.8)	26.6 (24.7, 29.8)	26.2 (24.3, 28.9)
**PSA (ng/mL), median (IQR)**	5.8 (4.5, 8.5)	6.0 (4.4, 8.6)	6.0 (4.6, 9.1)	5.5 (4.3, 8.0)	5.7 (4.5, 8.2)	5.7 (4.5, 8.1)	6.0 (4.5, 8.8)	5.8 (4.6, 8.4)	5.9 (4.5, 9.0)	5.5 (4.3, 7.8)	5.4 (4.4, 8.1)
**Calories (kcal), median (IQR)**	1914 (1528, 2355)	1631 (1266, 2000)	1806 (1454, 2261)	1856 (1508, 2280)	2055 (1653, 2517)	2277 (1900, 2733)	2300 (1942, 2723)	1953(1570, 2389)	18267 (144, 2279)	1748 (1429, 2199)	1727 (1386, 2116)
**Alcohol intake (servings per day), median (IQR)**	0.4 (0.0, 1.1)	0.3 (0.0, 1.1)	0.3 (0.0, 1.1)	0.4 (0.0, 1.2)	0.4 (0.0, 1.1)	0.5 (0.0, 1.2)	0.2 (0.0, 1.0)	0.2 (0.0, 1.1)	0.4 (0.0, 1.2)	0.4 (0.0, 1.3)	0.6 (0.1, 1.3)
**Wine intake (servings per day), median (IQR)**	0.1 (0.0, 0.3)	0.0 (0.0, 0.1)	0.1 (0.0, 0.3)	0.1 (0.0, 0.3)	0.1 (0.0, 0.4)	0.1 (0.0, 0.8)	0.0 (0.0, 0.1)	0.0 (0.0, 0.1)	0.1 (0.0, 0.3)	0.1 (0.0, 0.5)	0.1 (0.0, 0.8)
**Race[a], N (%)**											
African American	72 (3)	11 (2)	17 (4)	12 (3)	23 (4)	9 (2)	11 (2)	13 (3)	16 (3)	17 (5)	15 (3)
Asian/Pacific Islander	7 (0.3)	1 (0.2)	1 (0.2)	2 (0.5)	2 (0.4)	1 (0.3)	0 (0)	3 (1)	1 (0.2)	0 (0)	3 (1)
Latino	9 (0.4)	1 (0.2)	7 (2)	0 (0)	1 (0.2)	0 (0)	1 (0.2)	0 (0)	2 (0.4)	4 (1)	2 (0.5)
Mixed	3 (0.1)	0 (0)	1 (0.2)	0 (0)	2 (0.4)	0 (0)	0 (0)	1 (0.2)	1 (0.2)	0 (0)	1 (0.2)
Native American	2166 (95)	481 (97)	429 (94)	388 (96)	510 (94)	358 (96)	450 (97)	443 (96)	518 (95)	337 (94)	418 (93)
White	4 (0.2)	1 (0.2)	1 (0.2)	1 (0.3)	1 (0.2)	0 (0)	1 (0.2)	0 (0)	2 (0)	0 (0)	1 (0.2)
Unknown	72 (3)	11 (2)	17 (4)	12 (3)	23 (4)	9 (2)	11 (2)	13 (3)	16 (3)	17 (5)	15 (3)
**Smoking Status, N (%)**											
Never	1014 (45)	195 (40)	194 (43)	182 (46)	246 (46)	197 (54)	191 (42)	214 (47)	241 (45)	142 (40)	226 (51)
Former	1119 (50)	260 (53)	237 (53)	193 (48)	274 (51)	155 (42)	234 (51)	212 (47)	265 (50)	201 (57)	207 (47)
Current	112 (5)	35 (7)	20 (4)	23 (6)	19 (4)	15 (4)	33 (7)	29 (6)	28 (5)	10 (3)	12 (3)
**Walking Pace (mph), N (%)**											
Unable	27 (1)	10 (2)	6 (1)	6 (2)	3 (1)	2 (1)	4 (1)	8 (2)	5 (1)	6 (2)	4 (1)
Easy (<2)	360 (16)	83 (17)	88 (20)	54 (14)	85 (16)	50 (14)	97 (22)	78 (17)	88 (17)	50 (14)	47 (11)
Normal (2 to <3)	1116 (50)	252 (52)	229 (51)	216 (54)	248 (47)	171 (47)	232 (51)	242 (54)	261 (49)	192 (54)	189 (42)

© 2024 Liu VN et al. *JAMA Network Open.*

Brisk (3 to <4)	633 (28)	124 (26)	113 (25)	105 (26)	175 (33)	116 (32)	102 (23)	109 (24)	154 (29)	97 (27)	171 (38)
Fast (≥4)	92 (4)	12 (2)	14 (3)	18 (5)	20 (4)	28 (8)	16 (4)	12 (3)	21 (4)	9 (3)	34 (8)
**Family History of PCa, N (%)**											
No	1807 (79)	406 (82)	366 (80)	319 (79)	424 (78)	292 (78)	384 (83)	365 (79)	412 (76)	296 (82)	350 (78)
Yes	467 (21)	92 (18)	91 (20)	85 (21)	119 (22)	80 (22)	80 (17)	95 (21)	131 (24)	63 (18)	98 (22)
**Diabetes status, N (%)**											
No	2109 (93)	429 (86)	419 (92)	383 (95)	513 (94)	365 (98)	433 (93)	428 (93)	500 (92)	329 (92)	419 (94)
Yes	165 (7)	69 (14)	38 (8)	21 (5)	30 (6)	7 (2)	31 (7)	32 (7)	43 (8)	30 (8)	29 (6)
**Gleason at Diagnosis, N (%)**											
<7	1474 (66)	327 (67)	297 (66)	273 (68)	347 (65)	230 (63)	302 (67)	309 (68)	346 (65)	217 (61)	300 (67)
7	590 (26)	128 (26)	112 (25)	93 (23)	149 (28)	108 (30)	108 (24)	106 (23)	148 (28)	111 (31)	117 (26)
>7	171 (8)	31 (6)	42 (9)	33 (8)	37 (7)	28 (8)	41 (9)	40 (9)	36 (7)	26 (7)	28 (6)
**T-Stage at Diagnosis, N (%)**											
≤T1	1246 (55)	274 (55)	237 (52)	220 (54)	299 (55)	216 (58)	245 (53)	252 (55)	297 (55)	189 (53)	263 (59)
T2	997 (44)	218 (44)	213 (47)	180 (45)	235 (43)	151 (41)	210 (45)	200 (43)	240 (44)	169 (47)	178 (40)
T3a	31 (1)	6 (1)	7 (2)	4 (1)	9 (2)	5 (1)	9 (2)	8 (2)	6 (1)	1 (0.3)	7 (2)
**Primary Treatment, N (%)**											
Radical Prostatectomy	1377 (62)	307 (63)	268 (60)	244 (62)	318 (61)	240 (67)	271 (59)	284 (63)	328 (62)	216 (62)	278 (65)
Active Surv./Watchful Waiting	150 (7)	19 (4)	28 (6)	34 (9)	40 (8)	29 (8)	24 (5)	13 (3)	41 (8)	24 (7)	48 (11)
RT/Brachytherapy	444 (20)	105 (21)	97 (22)	82 (21)	102 (20)	58 (16)	99 (22)	103 (23)	104 (20)	70 (20)	68 (16)
Hormone Therapy	150 (7)	37 (8)	34 (8)	16 (4)	43 (8)	20 (6)	38 (8)	37 (8)	30 (6)	24 (7)	21 (5)
Other	87 (4)	22 (4)	18 (4)	18 (5)	18 (3)	11 (3)	25 (5)	12 (3)	26 (5)	12 (3)	12 (3)
**Multivitamin use, N (%)**											
Never	467 (21)	104 (21)	106 (24)	80 (20)	98 (18)	79 (21)	102 (23)	109 (24)	122 (23)	69 (19)	65 (15)
Former	444 (20)	107 (22)	84 (19)	76 (19)	105 (20)	72 (19)	84 (19)	92 (20)	111 (21)	73 (21)	84 (19)
Current	1329 (59)	278 (57)	259 (58)	240 (61)	333 (62)	219 (59)	266 (59)	252 (56)	305 (57)	214 (60)	292 (66)

Abbreviations: PDI, Overall Plant-based Diet Index; hPDI, Healthful Plant-based Diet Index; IQR, Interquartile Range; BMI, Body Mass Index; PSA, Prostate Specific Antigen; PCa, Prostate Cancer

[a]These are category labels participants were presented with on the survey (survey year: 2004).

© 2024 Liu VN et al. *JAMA Network Open.*

eTable 2: Hazard ratios and 95% confidence intervals[a] for multi-variable associations between each of the three food groups (sub-indices of the plant-based indices)[b] and risk of prostate cancer progression.

	**Prostate Cancer Progression**					
**Food Group**	Quintile 1 (lowest)	Quintile 2	Quintile 3	Quintile 4	Quintile 5 (highest)	P trend
Healthful Plant	1 (Reference)	0.86 (0.55, 1.34)	0.82 (0.54, 1.23)	0.91 (0.57, 1.44)	0.58 (0.34, 1.00)	0.08
Unhealthful Plant	1 (Reference)	0.96 (0.60, 1.54)	0.83 (0.52, 1.30)	1.18 (0.78, 1.78)	0.87 (0.41, 1.82)	0.93
Animal	1 (Reference)	1.18 (0.75, 1.85)	1.54 (0.80, 2.98)	1.35 (0.87, 2.11)	1.27 (0.70, 2.28)	0.30

[a]Models were adjusted for days diagnosed to first questionnaire (continuous), age diagnosed (continuous), year diagnosed (continuous), total energy intake (continuous, kcal/day), CaPSURE clinical site, T-stage at diagnosis (T1, T2, T3a), Gleason score at diagnosis (<7, 7, >7), PSA at diagnosis (≤6ng/mL, >6 to 10ng/mL, >10ng/mL); primary treatment (radical prostatectomy, radiation, hormonal therapy, watchful waiting/active surveillance, other); self-reported race (White, other (African American, Asian/Pacific Islander, Latino, Mixed, Native American, White, Unknown)); smoking status (current, former, never); walking pace (<2mph, 2 to <3mph, 3 to <4 mph, >4mph, unable), and BMI (continuous).
[b]The exposure groups were created by the sum of the food groups with positive scores for that group, i.e., healthful food group=sum of the seven food groups labeled as healthful; unhealthful food group=sum of the five food groups labeled as unhealthful; animal food group=sum of the six food groups labeled as animal).

eTable 3: Hazard ratios and 95% confidence intervals[a] for multi-variable associations between the healthy plant-based diet index and risk of prostate cancer progression among 2072 men initially diagnosed with non-metastatic prostate cancer, stratified by Gleason grade.

		**Healthful Plant-based Diet Index (hPDI)**					
		Quintile 1 (lowest)	Quintile 2	Quintile 3	Quintile 4	Quintile 5 (highest)	P trend
**Gleason grade**[b]							
<7 (N = 1375)	Events = 102	24	21	27	11	19	
	HR (95 CI)[b]	1 (Reference)	0.79 (0.58, 1.06)	1.04 (0.60, 1.81)	0.77 (0.40, 1.51)	1.23 (0.74, 2.07)	0.60
≥7 (N = 680)	Events = 86	26	21	19	9	11	
	HR (95 CI)[b]	1 (Reference)	0.81 (0.48, 1.39)	0.84 (0.58, 1.22)	0.68 (0.34, 1.35)	0.45 (0.25, 0.81)	0.01

Abbreviations: hPDI, Healthful Plant-based Diet Index; HR, Hazard Ratio; CI, Confidence Interval
[a]Models were adjusted for days diagnosed to first questionnaire (continuous), age diagnosed (continuous), year diagnosed (continuous), total energy intake (continuous, kcal/day), CaPSURE clinical site, T-stage at diagnosis (T1, T2, T3a), Gleason score at diagnosis (<7, 7, >7), PSA at diagnosis (≤6ng/mL, >6 to 10ng/mL, >10ng/mL); primary treatment (radical prostatectomy, radiation, hormonal therapy, watchful waiting/active surveillance, other); self-reported race (White, other (African American, Asian/Pacific Islander, Latino, Mixed, Native American, White, Unknown)); smoking status (current, former, never); walking pace (<2mph, 2 to <3mph, 3 to <4 mph, >4mph, unable), and BMI (continuous).
[b]p-interaction: 0.03

© 2024 Liu VN et al. *JAMA Network Open.*
