# Supplementary material for: Plant-Based Diets and Disease Progression in Men With Prostate Cancer
Source: JAMA Netw Open. 2024 May 1;7(5):e249053. doi: 10.1001/jamanetworkopen.2024.9053 (PMC11063803; doi:10.1001/jamanetworkopen.2024.9053)
Supplement: Supplement 2. — Data Sharing Statement [file jamanetwopen-e249053-s002.pdf]

Data Sharing Statement

Liu. Plant-Based Diets and Disease Progression in Men With Prostate Cancer. *JAMA Netw Open*. Published May 01, 2024. doi:10.1001/jamanetworkopen.2024.9053

Data
Data available: No

Additional Information
Explanation for why data not available: The patients and sites did not consent to share patient-level data publicly.
